# Comparison of diffusion weighted imaging b0 with T2*-weighted gradient echo or susceptibility weighted imaging for intracranial hemorrhage detection after reperfusion therapy for ischemic stroke

**DOI:** 10.1007/s00234-023-03180-3

**Published:** 2023-06-29

**Authors:** Sven P. R. Luijten, Nadinda A. M. van der Ende, Sandra A. P. Cornelissen, Leo Kluijtmans, Antonius van Hattem, Geert Lycklama a Nijeholt, Alida A. Postma, Reinoud P. H. Bokkers, Lars Thomassen, Ulrike Waje-Andreassen, Nicola Logallo, Serge Bracard, Benjamin Gory, Bob Roozenbeek, Diederik W. J. Dippel, Aad van der Lugt

**Affiliations:** 1 Department of Radiology and Nuclear Medicine, Erasmus University Medical Center, Rotterdam, The Netherlands; 2 Department of Neurology, Erasmus University Medical Center, Rotterdam, The Netherlands; 3 Department of Radiology, Isala Hospital, Zwolle, The Netherlands; 4 Department of Radiology, Haaglanden Medisch Centrum, Hague, The Netherlands; 5 Department of Radiology, Maastricht University Medical Center, Maastricht, The Netherlands; 6 Department of Radiology, Medical Imaging Center, University Medical Center Groningen, University of Groningen, Groningen, The Netherlands; 7 Department of Neurology, Haukeland University Hospital, Bergen, Norway; 8 Department of Neurosurgery, Haukeland University Hospital, Bergen, Norway; 9 Department of Diagnostic and Interventional Neuroradiology, University Hospital of Nancy, Nancy, France

**Keywords:** Ischemic stroke, Reperfusion, MRI, Intracranial hemorrhage

## Abstract

**Purpose:**

Diffusion-weighted imaging (DWI) b0 may be able to substitute T2*-weighted gradient echo (GRE) or susceptibility-weighted imaging (SWI) in case of comparable detection of intracranial hemorrhage (ICH), thereby reducing MRI examination time. We evaluated the diagnostic accuracy of DWI b0 compared to T2*GRE or SWI for detection of ICH after reperfusion therapy for ischemic stroke.

**Methods:**

We pooled 300 follow-up MRI scans acquired within 1 week after reperfusion therapy. Six neuroradiologists each rated DWI images (b0 and b1000; b0 as index test) of 100 patients and, after a minimum of 4 weeks, T2*GRE or SWI images (reference standard) paired with DWI images of the same patients. Readers assessed the presence of ICH (yes/no) and type of ICH according to the Heidelberg Bleeding Classification. We determined the sensitivity and specificity of DWI b0 for detection of any ICH, and the sensitivity for detection of hemorrhagic infarction (HI1 & HI2) and parenchymal hematoma (PH1 & PH2).

**Results:**

We analyzed 277 scans of ischemic stroke patients with complete image series and sufficient image quality (median age 65 years [interquartile range, 54–75], 158 [57%] men). For detection of any ICH on DWI b0, the sensitivity was 62% (95% CI: 50–76) and specificity 96% (95% CI: 93–99). The sensitivity of DWI b0 was 52% (95% CI: 28–68) for detection of hemorrhagic infarction and 84% (95% CI: 70–92) for parenchymal hematoma.

**Conclusion:**

DWI b0 is inferior for detection of ICH compared to T2*GRE/SWI, especially for smaller and more subtle hemorrhages. Follow-up MRI protocols should include T2*GRE/SWI for detection of ICH after reperfusion therapy.

**Supplementary Information:**

The online version contains supplementary material available at 10.1007/s00234-023-03180-3.

## Introduction

Intracranial hemorrhage (ICH) after reperfusion therapy for ischemic stroke is associated with poor clinical outcome and is an important safety endpoint in reperfusion trials [1–3]. Standard imaging protocols for follow-up with magnetic resonance imaging (MRI) typically include T2*-weighted gradient echo (GRE) or susceptibility-weighted imaging (SWI) sequences which are more sensitive for detection of ICH compared to non-contrast CT [4, 5].

Diffusion-weighted imaging (DWI) is also routinely acquired for detection of acute infarcts using b1000 images. Next to acquisition of b1000 images, clinical brain DWI sequences additionally include acquisition of b0 images without diffusion gradients that are T2*-weighted and, similar to T2*GRE/SWI images, sensitive to susceptibility effects caused by blood breakdown products [6]. Previous studies have compared the sensitivity of DWI b0 with T2*GRE images for detection of ICH and found inconsistent results. Some studies found that DWI b0 was inferior compared to T2*GRE or SWI for detection of ICH[7, 8], while another study found no difference [9]. These conflicting findings may have resulted from performing MRI at different magnetic field strengths (1.5 T or 3 T), and inclusion of hemorrhages with varying etiologies such as acute intracerebral hematomas, hemorrhagic transformation, and chronic cerebral microbleeds, as well as small numbers of hemorrhages. Hence, it is poorly understood whether DWI b0 offers similar detectability of ICH as T2*GRE or SWI following reperfusion therapy for ischemic stroke.

If the sensitivity of DWI b0 is similar to T2*GRE or SWI for detection of ICH then DWI may serve for evaluating both acute infarcts and presence of ICH without the need for additional acquisition of T2*GRE or SWI. This could result in reducing MRI examination time, which is desirable given the prolonged examination time compared to CT and the limited availability of MR scanners.

In this study, we evaluated the diagnostic accuracy of DWI b0 compared to T2*GRE or SWI for detection of ICH after reperfusion therapy for ischemic stroke.

## Methods

### Study population

This study was performed in accordance with the STARD guidelines for reporting diagnostic accuracy [10]. We pooled 300 MRI scans from two ischemic stroke trials, NORTEST (*n* = 112; Sept 2012 – Sept 2016) and THRACE (*n* = 90; June 2010 – Feb 2015), and a local cohort of ischemic stroke patients (*n* = 98; Sept 2019 – March 2021). NORTEST was a randomized controlled trial comparing the safety and efficacy of tenecteplase versus alteplase in patients eligible for intravenous thrombolysis (IVT) [11]. THRACE was a randomized controlled trial comparing the efficacy of endovascular thrombectomy (EVT) in addition to IVT versus IVT alone in patients with ischemic stroke due to anterior circulation intracranial large vessel occlusion (LVO) [12]. The local cohort consisted of patients undergoing EVT with or without IVT for ischemic stroke due to anterior circulation intracranial LVO. All MRI scans used in the present study were performed within 1 week after reperfusion therapy for ischemic stroke. We balanced the proportion of ICH-positive and ICH-negative examinations (1:1 ratio) based on prior assessments done by local radiologists. All patients or their legal representatives provided written informed consent for use of clinical and imaging data.

## MRI acquisition

Within each cohort, follow-up stroke MRI protocols included a DWI sequence for assessment of cerebral ischemia and either a T2*GRE or SWI sequence for assessment of ICH. In NORTEST and THRACE, follow-up imaging was performed at multiple sites with different MRI protocols resulting in varying acquisition parameters. In the local cohort, MRI was carried out with fixed acquisition parameters. An overview of scan acquisition parameters used within each cohort is provided in Supplementary Table 1.

## Image assessment

**Fig. 1:**
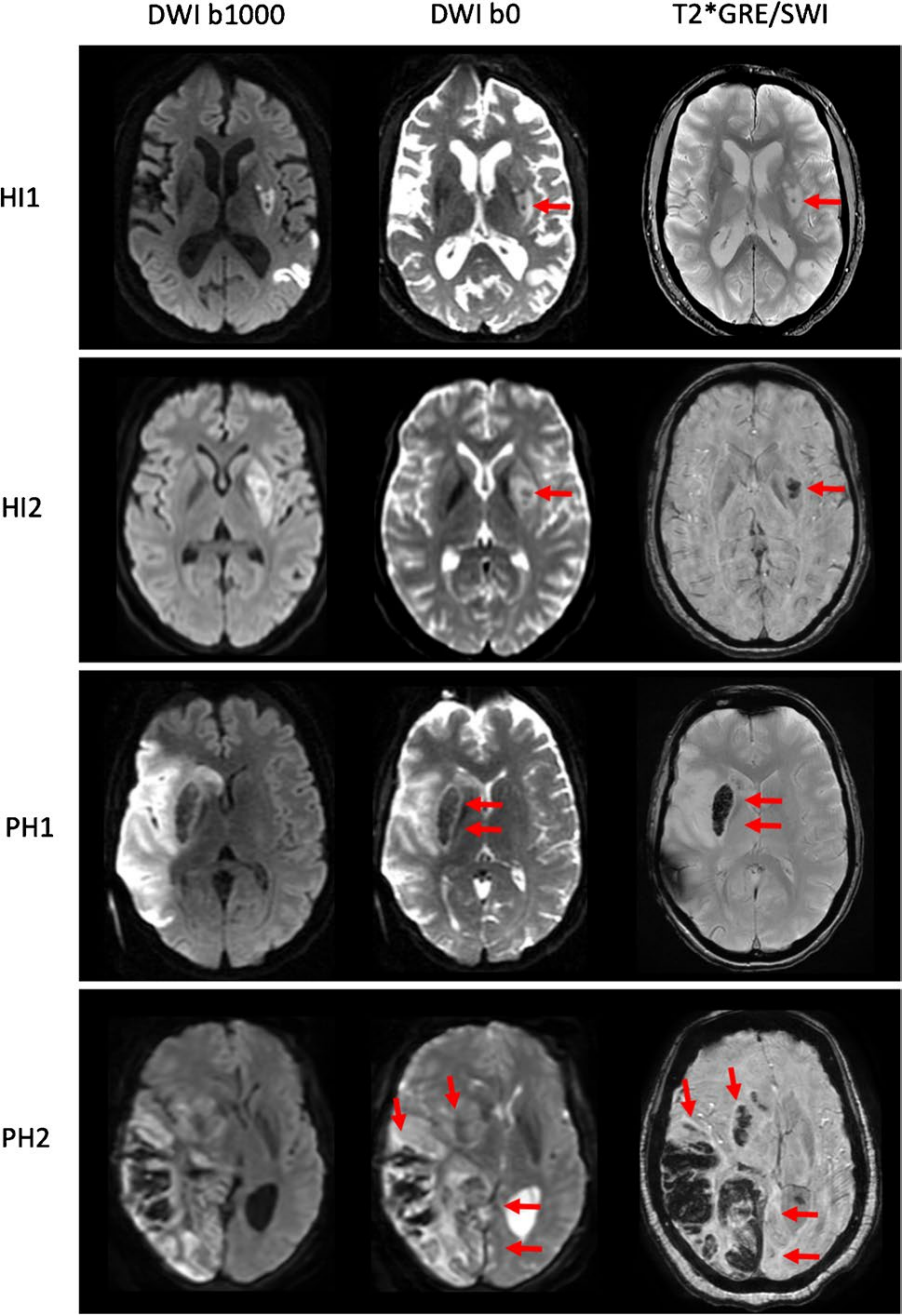
Detection of intracranial hemorrhage (ICH) on diffusion-weighted imaging (DWI) b0 and T2*GRE/SWI. Axial slices of four examinations are displayed from top to bottom showing HI1, HI2, PH1, and PH2 types of ICH indicated by the red arrows

Six neuroradiologists each read 100 pairs of DWI and T2*GRE/SWI images of the same patients. As a result, each pair of images was read by 2 neuroradiologists. First, each neuroradiologist received the DWI images (b0 and b1000) along with the corresponding ADC maps. Then, after a minimum of 4 weeks, to prevent recollection bias, each neuroradiologist received the paired T2*GRE/SWI images of the same patients along with the DWI (b0 and b1000) and ADC maps. For both T2*GRE/SWI and DWI b0 the MR signal of ICH in the hyper acute stage (24 h) is high, in the subsequent acute (1–3 days) and early subacute stage (3–7 days) it is low, in the late subacute stage (7–28 days) it is high again, and in the chronic stage (> 1 month) signal characteristics are variable. Assessments done on DWI b0 images served as the index test and were compared to assessments done on T2*GRE/SWI images served as the reference standard. The presence of any ICH (yes/no) and the type of ICH was assessed according to the Heidelberg Bleeding Classification (Table 1) [13]. Before conducting the assessments, all readers received a training session in which the scoring forms designed for the present study were explained. Additionally, all readers were provided with a document including guidelines, definitions, and examples of ICH types on DWI b0 and corresponding T2*GRE/SWI images of the same patients (Fig. 1). These examples were solely used for training and were not taken from the cohort used for analysis. Each reader was blinded for assessments done by other readers and for all clinical information except for the suspected location of the infarct (left or right hemisphere, or brainstem/cerebellum).

**Table 1:** Types of ICH and definitions according to the Heidelberg Bleeding Classification

Type	Description
Class 1. Hemorrhagic transformation of infarcted brain tissue	
HI1	Scattered small petechiae, no mass effect
HI2	Confluent petechiae, no mass effect
PH1	Hematoma within infarcted tissue, occupying < 30%, no substantive mass effect
Class 2. Intracerebral hemorrhage within and beyond infarcted brain tissue	
PH2	Hematoma occupying 30% or more of the infarcted tissue, with obvious mass effect
Class 3. Intracerebral hemorrhage outside the infarcted brain tissue or intracranial-extracranial hemorrhage	
rPH	Parenchymal hematoma remote from infarcted brain tissue
IVH	Intraventricular hemorrhage
SAH	Subarachnoid hemorrhage
SDH	Subdural hemorrhage

*HI*, hemorrhagic infarction, *PH*, parenchymal hematoma

## Statistical analysis

We estimated the sensitivity and specificity for detection of any ICH on DWI b0 compared to T2*GRE/SWI as the reference standard per reader and among all readers. In addition, we estimated the sensitivity for detection of hemorrhagic infarction (HI1 and HI2) and parenchymal hematoma (PH1 and PH2) among all readers. We did not estimate specificity for hemorrhagic infarction and parenchymal hematoma as this would not reflect true negative detection. This is because the group of patients negative for hemorrhagic infarction is a composite of patients with parenchymal hematoma and without any ICH, and the other way around (patients negative for parenchymal hematoma is a composite of patients with hemorrhagic infarction and without any ICH). Our study design created correlations from readers evaluating the same cases and correlations from readers and cases being paired across the reading conditions. Ignoring potential positive correlations caused by this design can lead to misleadingly narrow confidence intervals (CIs) [14]. To account for this, we constructed random-effects logistic regression models and considered readers and cases to be cross-correlated random effects when estimating CIs for sensitivity and specificity among all readers. Next, we studied the possible influence of magnetic field strength and use of different imaging sequences for the reference reading on the sensitivity and specificity of DWI b0 for detection of ICH. In order to do so, we estimated the overall sensitivity and specificity for detection of any ICH after stratifying scans based on magnetic field strength (1.5T versus 3T) and imaging sequence used for the reference reading (T2*GRE versus SWI). Finally, we determined the agreement among readers for detection of any ICH on DWI b0 using Cohen’s Kappa. For agreement regarding classification of ICH according to the Heidelberg Bleeding Classification (no ICH, hemorrhagic infarction type 1 [HI1], hemorrhagic infarction type 2 [HI2], parenchymal hematoma type 1 [PH1], parenchymal hematoma type 2 [PH2]) on DWI b0 we used Cohen’s weighted Kappa taking into account the degree of disagreement. Statistical analyses were done in R (version 4.1.1) using the packages lme4, epiR, and vcd.

## Results

We included MRI scans from a total of 277 ischemic stroke patients (median age: 65 years [IQR: 54 – 75 years]; 158 men [57.0%]; Table 2). We excluded 5 patients due to patient motion during MRI and 18 patients due to incomplete DWI series. Median time between stroke onset and performing MRI was 26 h (IQR: 22–34 h), 105 MRIs (37.9%) were performed at 1.5 T and 172 (62.1%) at 3 T, and in 107 examinations (38.6%) a T2*GRE sequence was used for ICH detection and in 170 examinations (61.4%) a SWI sequence.

**Table 2:** Baseline characteristics

	Total study population (*n* = 277)
Age, years	65 (54–75)
Male sex	158 (57.0%)
Baseline NIHSS	12 (6–19)
IVT	227 (81.9%)
EVT	120 (43.3%)
LVO*	200 (72.2%)
Stroke onset to MRI, hours	26 (22 – 34)
Magnetic field strength	
1.5 T	105 (37.9%)
3 T	172 (62.1%)
Sequence used as reference	
T2*GRE	107 (38.6%)
SWI	170 (61.4%)

Data are presented as count (%) or median (IQR)

^*^LVO includes occlusion of the intracranial carotid artery (ICA) or ICA terminus (ICA-T), M1 segment of the middle cerebral artery (MCA), and proximal M2-MCA

*NIHSS*, National Institutes of Health Stroke Scale; *IVT*, intravenous thrombolysis; *EVT*, endovascular thrombectomy; *LVO*, large vessel occlusion; *GRE*, gradient recalled echo; *SWI*, susceptibility weighted imaging

## Comparison of ICH detection

Sensitivity and specificity of DWI b0 compared to T2*GRE/SWI for detection of ICH is summarized for each reader in Table 3. Among a total of 546 paired readings which included assessments of DWI b0 and T2*GRE or SWI images of the same patients done by the six readers, presence of ICH was detected for 277 reads (50.7%) and absence of ICH in for 269 reads (49.3%) on T2*GRE/SWI. In comparison, on DWI b0, ICH presence was correctly detected for 172 reads (31.5%) and absence of ICH for 259 reads (47.4%; Supplementary tables 3a-f). This resulted in an overall sensitivity of 62% (95% CI: 50–76) and specificity of 96% (95% CI: 94–99; Table 3) for detection of any ICH on DWI b0. When stratifying by ICH type, we found a sensitivity of 52% (95% CI: 28–68) for detection of hemorrhagic infarction (HI1 and HI2), and a sensitivity of 84% (95% CI: 70–92) for detection of parenchymal hematoma (PH1 and PH2).

**Table 3:** Sensitivity and specificity of DWI b0 for detection of ICH per reader and among all readers

	ICH present/ ICH absent*	Sensitivity (95% CI)	Specificity (95% CI)
Reader 1	45 / 45	58% (42 – 72)	100% (92 – 100)
Reader 2	44 / 47	57% (41 – 71)	92% (80 – 98)
Reader 3	46 / 49	77% (62 – 89)	91% (80 – 98)
Reader 4	47 / 43	53% (38 – 68)	95% (84 – 99)
Reader 5	46 / 41	63% (48 – 77)	100% (91 – 100)
Reader 6	49 / 44	65% (50 – 78)	100% (92 – 100)
Overall	277 / 269	62% (50 – 76)	96% (94 – 99)

^*^According to reference reading on T2*GRE/SWI

**Fig. 2:**
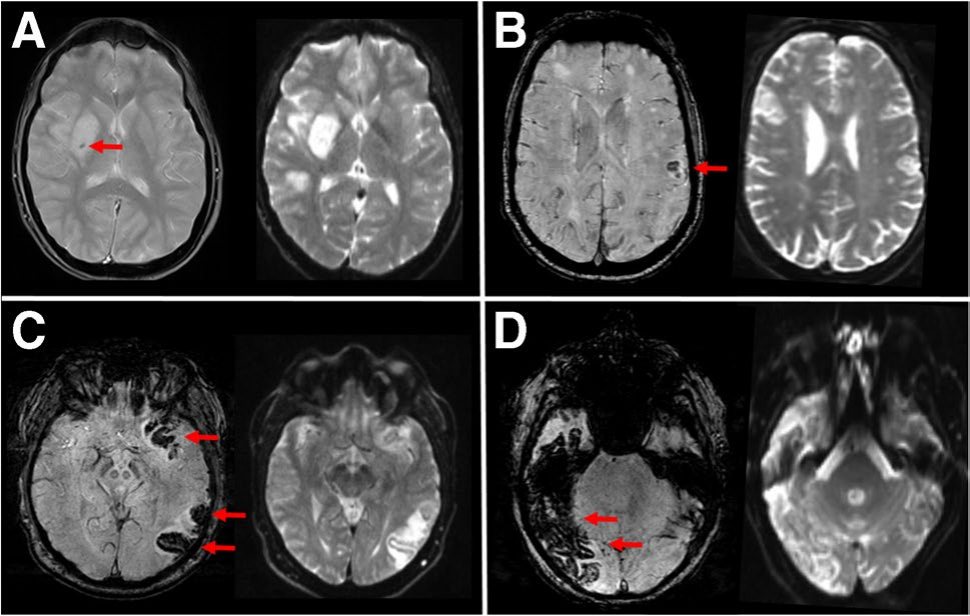
Different types of intracranial hemorrhages indicated by red arrows on T2*GRE/SWI but missed on DWI b0. (**A**) Small petechial hemorrhage classified as HI1 on T2*GRE at 1.5T but missed on DWI b0. (**B**) Small confluent hemorrhages classified as HI2 on SWI at 3T but missed on DWI b0. (**C**) Parenchymal hematoma classified as PH1 on SWI at 1.5T but missed on DWI b0. (**D**) Large parenchymal hematoma classified as PH2 on SWI at 1.5T but missed on DWI b0

On DWI b0, a total of 127 ICHs (42.4%) were missed compared to T2*GRE/SWI. To explore which types of hemorrhages were frequently missed, we determined the proportion of missed hemorrhages on DWI b0 compared to T2*GRE/SWI according to ICH type (Table 4; Fig. 2). We found that HI1 (68%) and hemorrhages outside of infarcted brain tissue including parenchymal hematoma remote from infarcted brain tissue (rPH, 100%), intraventricular hemorrhage (IVH, 73%), subarachnoid hemorrhage (SAH, 88%), and subdural hemorrhage (SDH, 50%) were missed in a substantial number of reads.

**Table 4:** Type of ICH detected on T2*GRE/SWI and proportion missed on DWI b0

Type	No. detected on T2*GRE/SWI	No. missed on DWI b0 (%)
Class I		
HI1	57	39 (68%)
HI2	96	35 (36%)
PH1	56	16 (29%)
Class II		
PH2	49	1 (2%)
Class III		
rPH	2	2 (100%)
IVH	15	11 (73%)
SAH	25	22 (88%)
SDH	2	1 (50%)
Total*	302	127 (42%)

^*^Total number of ICH exceeds 277 because multiple ICH types are present in a subgroup of patients (e.g., parenchymal hematoma type 2 + intraventricular hemorrhage)

*GRE*, gradient-recalled echo; *SWI*, susceptibility- weighted imaging; *DWI*, diffusion-weighted imaging; *HI*, hemorrhagic infarction; *PH*, parenchymal hematoma; *rPH*, remote parenchymal hematoma; *IVH*, intraventricular hemorrhage; *SAH*, subarachnoid hemorrhage; *SDH*, subdural hemorrhage

When imaging was performed at 3T, we found a sensitivity of 68% (95% CI: 56–84) and specificity of 95% (95% CI: 91–99) for detection of any ICH on DWI b0 compared to a sensitivity of 50% (95% CI: 33–64) and specificity of 98% (95% CI: 93–100) when imaging was performed at 1.5T. When using T2*GRE as the reference standard, we found a sensitivity 66% (95% CI: 52–79) and specificity of 97% (95% CI: 91–99) for detection of any ICH on DWI b0 compared to a sensitivity of 60% (95% CI: 43–77) and specificity of 96% (95% CI: 92–99) when using SWI as the reference standard. There was substantial agreement among readers for detection of any ICH on DWI b0 (Kappa 0.67, 95% CI: 0.57–0.76) and for classification of ICH according to the Heidelberg Bleeding Classification on DWI b0 (weighted Kappa 0.69, 95% CI: 0.61–0.77).

## Discussion

In this study, we found that DWI b0 is inferior for detection of ICH as compared to T2*GRE/SWI, particularly for detection of hemorrhagic infarction and less so for parenchymal hematoma. We further found that the detection of ICH on DWI b0 was negatively influenced when imaging was performed at 1.5T compared to 3T and when SWI was used for the reference reading compared to T2*GRE.

Our findings are in line with two previous studies, which demonstrated that the sensitivity of DWI b0 was inferior compared to T2*GRE when acquired at 1.5T. In contrast, these studies used MRI scans performed in patients presenting with suspected stroke symptoms including hemorrhages with varying etiologies such as acute intracerebral hematomas, hemorrhagic transformation, and chronic cerebral microbleeds. Therefore, it was still unclear what the diagnostic accuracy was of DWI b0 for detection of ICH after reperfusion therapy with IVT and EVT. We now show that DWI b0 is inferior for detection of ICH after reperfusion therapy when compared to T2*GRE and SWI sequences acquired at both 1.5T and 3T. Further stratifying hemorrhages according to the Heidelberg Bleeding Classification revealed that smaller and more subtle hemorrhages are more frequently missed than larger parenchymal hematomas. Another study found no difference between detection of ICH when using DWI b0 compared to T2*GRE. However, this study included only 9 patients with ICH and did not report different types of hemorrhage in detail [9].

Sensitivity of ICH detection on DWI b0 images acquired at 3T was higher than at 1.5T. This can be explained by the fact that susceptibility effects scale linearly with magnetic field strength thereby increasing image contrast and conspicuity of ICH [15, 16]. Since image contrast of DWI b0 is generally lower compared to T2*GRE/SWI images, the former likely benefits more from this increase. Conversely, the sensitivity of DWI b0 for detection of ICH was lower when using SWI compared to T2*GRE images as the reference standard. This is also to be expected because SWI is more sensitive compared to T2*GRE for smaller and more subtle hemorrhages [17, 18]. We show that such hemorrhages are more likely to be missed on DWI b0 resulting in a lower sensitivity of DWI b0 when compared to SWI versus T2*GRE. Additional differences in acquisition parameters between DWI b0 and T2*GRE/SWI sequences likely also influence image contrast and conspicuity of ICH. This includes parameters such as two dimensional versus three-dimensional image acquisition, echo time, and spatial resolution [19].

Our results clearly show that T2*GRE/SWI sequences should not be replaced by DWI b0 in follow-up stroke MRI protocols to assess ICH after reperfusion therapy. Especially when noting that not only hemorrhagic infarctions (HI1 and HI2) but also parenchymal hematomas (PH1 and PH2) were missed in a substantial number of cases. Furthermore, it has recently been shown that the parenchymal hematomas (PH1 and PH2) are associated with poor functional outcome and are therefore clinically important to detect [2, 3]. If available, SWI is preferred over T2*GRE due to improved detection smaller and subtle hemorrhages [17, 18]. Additionally, with the availability of phase encoding information, SWI also allows discriminating between different causes of susceptibility for instance between hemorrhage and calcification [19].

The main strength of this study is that we included scans acquired with various acquisition protocols reflective of clinical practice among different centers and countries. This made it possible to compare the sensitivity of DWI b0 to both T2*GRE and SWI and when acquired at different magnetic field strengths. In addition, this allows broad generalizability of the current findings. Some limitations must also be considered. First, we balanced the ratio of ICH-positive and ICH-negative cases in order to increase statistical precision but this does not reflect the prevalence of ICH after reperfusion therapy in routine clinical practice. Second, we included only a limited number of scans with rPH, IVH, SAH, and SDH, limiting the ability to compare detection of these hemorrhages on DWI b0 compared to T2*GRE/SWI. Third, the present findings are restricted to patients undergoing reperfusion therapy but not generalizable to other populations such as patients with primary acute intracerebral hematomas. Fourth, T2*GRE/SWI is imperfect as reference standard but chosen for obvious pragmatic reasons. Fifth, readers were potentially disadvantaged by not having additional imaging sequences available such as T1 and FLAIR imaging that may have improved hemorrhage detection. Sixth, DWI was acquired with routine clinical acquisition parameters used to depict infarction but not ICH. It is likely that further optimization of DWI acquisition e.g., by improving the spatial resolution, could lead to improved detection of smaller and more subtle hemorrhages. Lastly, we used scans acquired within a time window of 1 week after reperfusion therapy, without accounting for potential differences in signal characteristics of hemorrhages at different time points due to natural evolution [20]. However, since the vast majority of scans was acquired with 24–48 h after stroke onset, we find it unlikely that accounting for such differences in signal characteristics of hemorrhages will yield different results.

In conclusion, we found that DWI b0 is inferior compared to T2*GRE/SWI for detection of ICH after reperfusion therapy for ischemic stroke, especially for smaller and more subtle hemorrhages. Furthermore, the detection of ICH on DWI b0 was negatively influenced when acquired at lower magnetic field strength and when compared to SWI. Follow-up stroke MRI protocols should include T2*GRE/SWI sequences for detection of ICH after reperfusion therapy.

### Supplementary Information

Below is the link to the electronic supplementary material.

Supplementary file1 (DOCX 15 KB)
