# Supplementary material for: Comparison of diffusion weighted imaging b0 with T2*-weighted gradient echo or susceptibility weighted imaging for intracranial hemorrhage detection after reperfusion therapy for ischemic stroke
Source: Neuroradiology. 2023 Jun 29;65(11):1649–55. doi: 10.1007/s00234-023-03180-3 (PMC10567825; doi:10.1007/s00234-023-03180-3)
Supplement: Supplementary file 1 — Supplementary file1 (DOCX 15 KB) [file 234_2023_3180_MOESM1_ESM.docx]

**Supplemental material to:**

**Comparison of diffusion weighted imaging b0 with T2*-weighted gradient echo or susceptibility weighted imaging for intracranial hemorrhage detection after reperfusion therapy for ischemic stroke**

Sven P.R. Luijten, Nadinda A.M. van der Ende, Sandra A.P. Cornelissen, Leo Kluijtmans, Antonius van Hattem, Geert Lycklama a Nijeholt, Alida A. Postma, Reinoud P.H. Bokkers, Lars Thomassen, Ulrike Waje-Andreassen, Nicola Logallo, Serge Bracard, Benjamin Gory, Bob Roozenbeek, Diederik W.J. Dippel, Aad van der Lugt

**Supplementary Table 1.** Scan acquisition parameters per cohort

|  |  | **T2*GRE** | | | **SWI** | | | **DWI** | | |
| --- | --- | --- | --- | --- | --- | --- | --- | --- | --- | --- |
|  | **Field**  **strength** | **TR/TE**  **(ms)** | **Slice**  **Thickness (mm)** | **Pixel size**  **(mm^2^)** | **TR/TE**  **(ms)** | **Slice**  **Thickness (mm)** | **Pixel size**  **(mm^2^)** | **TR/TE**  **(ms)** | **Slice**  **Thickness (mm)** | **Pixel size**  **(mm^2^)** |
| **NORTEST** | 1.5T | 465-800/  20-50 | 1.5-5 | 0.50 x 0.50 –  0.90 x 0.90 | 27-89/  14-20 | 1.5-5 | 0.45 x 0.45 –  0.90 x 0.90 | 2460-8000/  56-99 | 3-5 | 0.90 x 0.90 –  1.88 x 1.88 |
|  | 3T | n/a | n/a | n/a | 27/20 | 1.5 | 0.86 x 0.86 | 2300-12000/  54-120 | 3-5 | 0.45 x 0.45 –  1.38 x 1.38 |
| **THRACE** | 1.5T | 440-950/  13-40 | 3-6 | 45 x 45 –  0.98 x 0.98 | n/a | n/a | n/a | 2700-9000/  70-114 | 3-6 | 0.60 x 0.60 –  1.95 x 1.95 |
|  | 3T | 520-900/  12-50 | 1.8-4 | 0.43 x 0.43 –  0.94 x 0.94 | n/a | n/a | n/a | 2500-12000/  55-114 | 3-5 | 0.81 x 0.81 –  1.80 x 1.80 |
| **Local cohort** | 1.5T | n/a | n/a | n/a | n/a | n/a | n/a | n/a | n/a | n/a |
|  | 3T | n/a | n/a | n/a | 91/48 | 1.8 | 0.49 x 0.49 | 8000/60 | 3 | 0.94 x 0.94 |

Abbreviations: GRE, gradient-recalled echo; SWI, susceptibility-weighted imaging; DWI, diffusion-weighted imaging; TR, repetition time; TE, echo time.

**Supplementary Table 2.** Crosstable with detection of ICH on DWI b0 (rows) versus T2*GRE/SWI (columns)

|  | **ICH+ on T2*GRE/SWI** | **ICH- on T2*GRE** | **Total** |
| --- | --- | --- | --- |
| **ICH+ DWI b0** | 172 | 10 | 182 |
| **ICH- DWI b0** | 105 | 259 | 364 |
| **Total** | 277 | 269 | 546 |

Abbreviations: ICH, intracranial hemorrhage; GRE, gradient-recalled echo; SWI, susceptibility-weighted imaging; DWI, diffusion-weighted imaging.

**Supplementary Table 3a.** Crosstable with detection of ICH on DWI b0 (rows) versus T2*GRE/SWI (columns) for reader 1.

|  | **ICH+ on T2*GRE/SWI** | **ICH- on T2*GRE/SWI** | **Total** |
| --- | --- | --- | --- |
| **ICH+ DWI b0** | 26 | 0 | 26 |
| **ICH- DWI b0** | 19 | 45 | 64 |
| **Total** | 45 | 45 | 90 |

Abbreviations: ICH, intracranial hemorrhage; GRE, gradient-recalled echo; SWI, susceptibility-weighted imaging; DWI, diffusion-weighted imaging.

**Supplementary Table 3b.** Crosstable with detection of ICH on DWI b0 (rows) versus T2*GRE/SWI (columns) for reader 2.

|  | **ICH+ on T2*GRE/SWI** | **ICH- on T2*GRE/SWI** | **Total** |
| --- | --- | --- | --- |
| **ICH+ DWI b0** | 34 | 4 | 38 |
| **ICH- DWI b0** | 10 | 43 | 53 |
| **Total** | 43 | 47 | 91 |

**Supplementary Table 3c.** Crosstable with detection of ICH on DWI b0 (rows) versus T2*GRE/SWI (columns) for reader 3.

|  | **ICH+ on T2*GRE/SWI** | **ICH- on T2*GRE/SWI** | **Total** |
| --- | --- | --- | --- |
| **ICH+ DWI b0** | 26 | 4 | 30 |
| **ICH- DWI b0** | 20 | 45 | 65 |
| **Total** | 46 | 49 | 95 |

**Supplementary Table 3d.** Crosstable with detection of ICH on DWI b0 (rows) versus T2*GRE/SWI (columns) for reader 4.

|  | **ICH+ on T2*GRE/SWI** | **ICH- on T2*GRE/SWI** | **Total** |
| --- | --- | --- | --- |
| **ICH+ DWI b0** | 25 | 2 | 27 |
| **ICH- DWI b0** | 22 | 41 | 63 |
| **Total** | 47 | 43 | 90 |

**Supplementary Table 3e.** Crosstable with detection of ICH on DWI b0 (rows) versus T2*GRE/SWI (columns) for reader 5.

|  | **ICH+ on T2*GRE/SWI** | **ICH- on T2*GRE/SWI** | **Total** |
| --- | --- | --- | --- |
| **ICH+ DWI b0** | 29 | 0 | 29 |
| **ICH- DWI b0** | 17 | 41 | 58 |
| **Total** | 46 | 41 | 87 |

**Supplementary Table 3f.** Crosstable with detection of ICH on DWI b0 (rows) versus T2*GRE/SWI (columns) for reader 6.

|  | **ICH+ on T2*GRE/SWI** | **ICH- on T2*GRE/SWI** | **Total** |
| --- | --- | --- | --- |
| **ICH+ DWI b0** | 32 | 0 | 32 |
| **ICH- DWI b0** | 17 | 44 | 61 |
| **Total** | 49 | 44 | 93 |
